# Podocyturia: A Clue for the Rational Use of Amiloride in Alport Renal Disease

**DOI:** 10.1155/2016/1492743

**Published:** 2016-01-28

**Authors:** H. Trimarchi, R. Canzonieri, A. Muryan, A. Schiel, A. Araoz, M. Paulero, J. Andrews, T. Rengel, M. Forrester, F. Lombi, V. Pomeranz, R. Iriarte, E. Zotta

**Affiliations:** ^1^Nephrology Service, Hospital Britanico de Buenos Aires, Perdriel 74, 1280 Buenos Aires, Argentina; ^2^Laboratory Service, Hospital Britanico de Buenos Aires, Perdriel 74, 1280 Buenos Aires, Argentina; ^3^IFIBIO Houssay, UBA CONICET, Facultad de Medicina, Universidad de Buenos Aires, Paraguay 2155, 1425 Buenos Aires, Argentina

## Abstract

No specific or efficient treatment exists for Alport syndrome, an X-linked hereditary disease caused by mutations in collagen type IV, a crucial component of the glomerular basement membrane. Kidney failure is usually a major complication of the disease, and patients require renal replacement therapy early in life. Microhematuria and subsequently proteinuria are hallmarks of kidney involvement, which are due to primary basement membrane alterations that mainly cause endothelial thrombosis and podocyte contraction and ulterior irreversible detachment. Commonly drug-based approaches include angiotensin-converting enzyme inhibitors and angiotensin receptor blockers, which are employed to reduce proteinuria and thus retard kidney disease progression and cardiovascular morbidity and mortality. However, as any hereditary disease, it is expressed as early as in the intrauterine life, and usually an index case is helpful to detect family-related cases. As no specific treatment exists, pathophysiologically based approaches are useful. The present case illustrates the reduction rate of urinary podocyte loss and proteinuria after amiloride administration and suggests the molecular pathways involved in Alport renal disease. Finally, podocyturia rather than proteinuria should be considered as an earlier biomarker of kidney involvement and disease progression in Alport disease.

## 1. Introduction

Alport syndrome is a chromosome X-linked hereditary disease with systemic involvement, mainly affecting the renal, pulmonary, visual, and auditory systems. It is due to different mutations in collagen IV [1]. Alport disease lacks specific therapy. Aims are directed to reducing the rate of progression of the organs involved, whenever possible. In this respect, chronic kidney disease is managed with the usual nephroprotective recommendations, as loss of weight, blood pressure control, salt restriction, and tobacco avoidance, amongst others. Angiotensin-converting enzyme inhibitors and angiotensin receptor blockers have been shown to be helpful to reduce kidney function decline, particularly when proteinuria exists [2]. Regrettably, only relatives linked to the index case take profit of these limited recommendations. All patients progress to end-stage kidney disease early in adulthood [1]. Therefore, early effective interventions in asymptomatic patients are mandatory. Urinary podocyte loss is a silent phenomenon that precedes proteinuria in glomerular diseases [3, 4]. Podocyturia is irreversible and any attempt to decrease its amount, particularly in early phases of a glomerulopathy, should be accompanied by reductions in proteinuria and delays in kidney function decline. We present a young male patient with a family history of kidney biopsy-proven Alport disease. He had normal kidney function, microhematuria, and mild proteinuria. His podocyturia was higher than age-matched controls. After amiloride prescription, his proteinuria turned negative and was detected as microalbuminuria, while podocyturia decreased to control rates. We suggest the potential roles certain integrins, the urokinase plasminogen activator (uPA), its receptor (uPAR), and plasmin may play in the pathogenesis of Alport kidney disease.

## 2. Case Presentation

A 25-year-old male with a family background of Alport disease was referred for an assessment of kidney involvement. Family history consisted of a mother-related uncle on dialysis with biopsy-proven Alport disease, another mother-related cousin with kidney involvement, and a grandfather who died due to sudden death at the age of 42. The patient was asymptomatic and normotensive (blood pressure 115/68 mmHg), with a body mass index of 24 and no tobacco consumption. Main laboratory results included hematocrit 44%, glycemia 78 mg/dL, serum creatinine 1.07 mg/dL, creatinine clearance of 74 mL/min, sodium 143 mEq/L, potassium 3.9 mEq/L, serum albumin 4.3 g/dL, and 24-hour urinary albumin excretion 250 mg/day. A renal ultrasound was normal.

**Figure 1:**
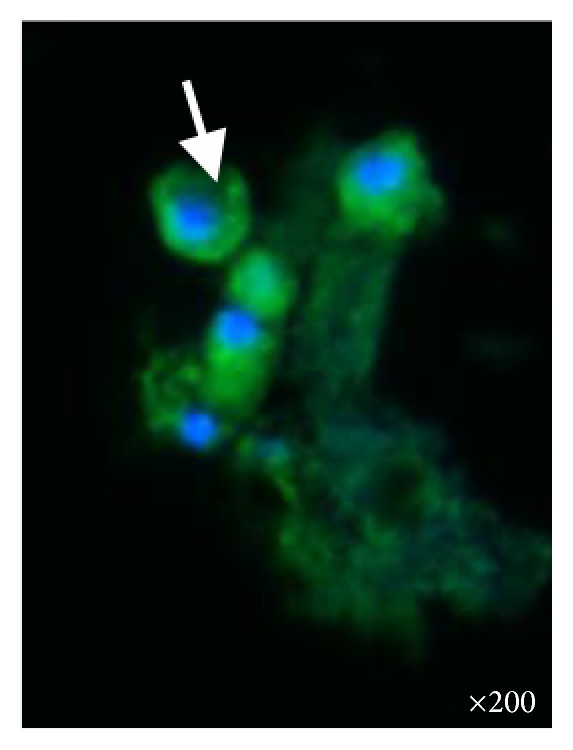
White arrow depicts numerous urinary podocytes appearing as bright green round fluorescent cells before amiloride in the patient with Alport syndrome. Fluorescent microscopy ×200.

**Figure 2:**
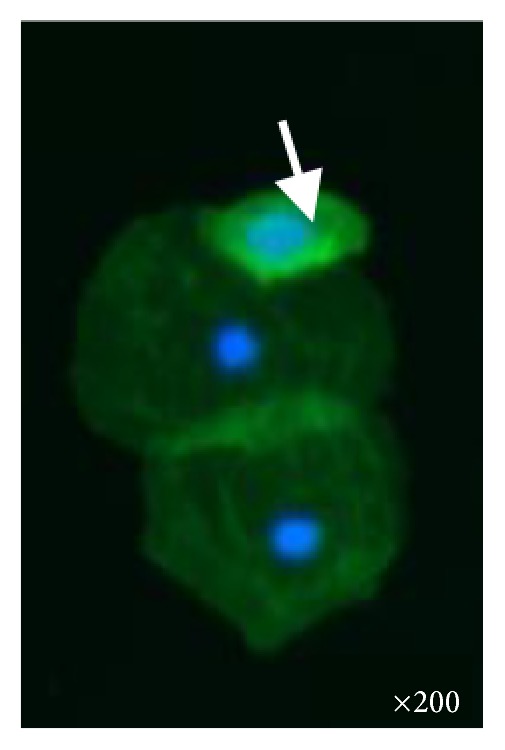
Urine specimen from a control. One podocyte (white arrow) and mainly tubular cells are observed. Fluorescent microscopy ×200.

**Figure 3:**
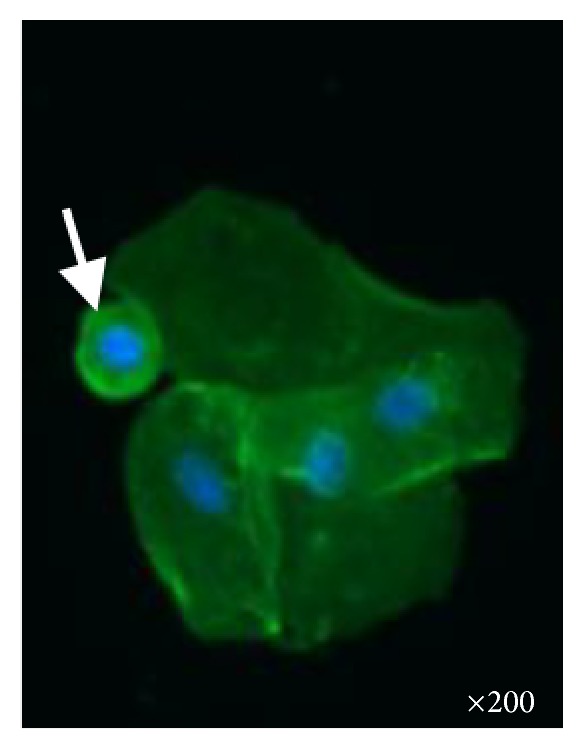
Urinary smear displays one podocyte after amiloride (white arrow). Fluorescent microscopy ×200.

Podocyturia was assessed as follows: fresh urine samples were centrifuged at 1500 rpm during 5 min and the supernatant was discarded; 10% formaldehyde in phosphate-buffered saline (PBS) pH 7.2–7.4 was added to the sediment to cover it. Smears were made from each sample on 2% silane-coated slides. The slides were stained with immunofluorescence technique and observed by epifluorescent microscopy. The slides were preincubated with no immune rabbit serum in phosphate-buffered saline 0.1 M, pH 7.4 (PBS, 1 : 100) at room temperature for 30 minutes, followed by incubation with a polyclonal anti-synaptopodin antibody (1 : 100, ab109560 Alexa Fluor^®^, Abcam, Cambridge, United Kingdom) overnight in a wet chamber at 4°C. After several rinses in PBS, the slides were incubated with anti-rabbit IgG secondary antibody (1 : 200 Alexa Fluor 488, Abcam, Cambridge, United Kingdom) for 2 hours at room temperature in a wet chamber. Finally, all the slides were mounted using Fluoroshield Mounting Medium with DAPI (Abcam, Cambridge, United Kingdom) and observed in an epifluorescent microscope (Nikon Eclipse E200, Nikon, Tokyo, Japan). Negative controls were performed without primary antibodies. Podocyte counting was assessed by counting in urinary smears the number of cells in 10 microscopy fields of ×20. The podocyte count was 1.07 cells per ×20 field; the number of podocytes per gram of urinary creatinine was 29.9, and the number of podocytes/100 mL of urine was 5.35 (Figure 1). This result was compared with 5 controls (Figure 2): 3 males and 2 females; mean age 22 ± 7.2 years with no past history of morbidities; creatinine clearance 108 mL/min; mean 24-hour urinary albumin excretion 88 ± 11 mg/day. The mean podocyte count was 0.12 ± 0.1 cells per ×20 field while the mean number of podocytes per gram of urinary creatinine was 10.7 and the mean number of podocytes/100 mL of urine was 1.1. The patient declined to undergo a kidney biopsy but accepted to receive amiloride 5 mg/day orally. After three months of therapy new podocyturia and laboratory results were obtained. The podocyte count was 0.2 cells per ×20 field; the number of podocytes per gram of urinary creatinine was 9, and the number of podocytes/100 mL of urine was 1 (Figure 3). Blood pressure was 110/70 mmHg, serum creatinine 1.09 mg/dL, creatinine clearance 79 mL/min, sodium 143 mEq/L, potassium 3.9 mEq/L, and 24-hour urinary albumin excretion 19 mg/day.

## 3. Discussion

In the present report, we demonstrate that amiloride may prove to be an efficient drug to decrease podocyturia and proteinuria in a patient with chronic kidney disease stage II with Alport syndrome.

Alport syndrome is an inherited progressive form of glomerular disease often associated with sensorineural hearing loss and ocular abnormalities [5–8]. The prevalence of the disease is calculated at approximately 1 in 5,000 live births and accounts for 0.3 to 2.3 percent of new cases of end-stage renal disease [9–11]. Alport syndrome is a primary basement membrane disorder due to mutations in genes encoding several members of type IV collagen. Mutations affecting the *α*-3, *α*-4, and *α*-5 chains of collagen IV impair their deposition into the collagen network, leading to secondary changes in the glomerular basement membrane composition and to the development of glomerulosclerosis [1]. The glomerular basement membrane specifically contains collagen type IV, and its structure is severely affected by the abnormal bundles of collagen. Glomerular endothelial cells and podocytes are constitutively attached to the membrane, and all three components function as a histologic and physiologic unit known as the glomerular filtration barrier. Podocytes continuously interact with the basement membrane, creating a permanent cross-talk between the podocyte cellular membrane molecules and the glomerular basement membrane components.

Initially, in Alport disease microhematuria first and proteinuria later on are considered to be mainly due to disruptions in the glomerular basement membrane [1, 2, 5–7]. However, proteinuria can also be caused by podocyte depletion in Alport syndrome, in agreement with our findings [12]. Although the molecular mechanisms of podocyte detachment in Alport disease have not been explored, our present report, showing an important reduction in podocyturia after amiloride prescription, may suggest that certain molecules could be involved in podocyte loss. This glomerular podocytopenia is critical as it impairs the normal glomerular filtration process and determines the sclerosis and fibrosis of the glomerulus and the irreversible route to end-stage renal disease [13, 14].

Plasma and urinary levels of plasminogen and plasmin are elevated in patients with proteinuria and nephrotic syndrome [15]. Plasminogen conversion to plasmin is in part mediated by urokinase-type plasminogen activator (uPA), an enzyme that binds to a specific plasma cell membrane receptor, uPAR [16]. In this regard, amiloride competitively inhibits the catalytic activity of u-PA, decreasing plasmin levels. In turn, plasmin has been implicated in the generation of oedema in glomerulopathies and in the nephrotic syndrome, activating ENa^+^C channels at the distal tubule [17]. Moreover, Zhang et al. have shown that amiloride also reduces uPAR expression and inhibits uPAR mRNA and protein synthesis in podocytes [18]. uPAR interacts with certain integrins that modulate ligand-binding actions [19]. Lipid raft-associated uPAR and *β*_3_ integrin, located at the basal compartment of the podocyte anchoring the cell to the basement membrane, form a complex causing the activation of the integrin. It is a key event that mediates uPAR-induced cellular events as actin-mediated podocyte contraction, slit diaphragm widening, increased motility, detachment, and proteinuria [18]. Some studies have also demonstrated the uPAR-*β*_3_ integrin signaling to be involved in the development of proteinuria [18, 20, 21]. We have previously reported a successful enduring antiproteinuric effect mediated by amiloride [22]. Noteworthily, uPAR is highly expressed on the cell surface of diseased podocytes, but only scarcely on normal podocytes [18].

Additionally, plasminogen activator inhibitor-1 (PAI-1) also presents pleiotropic actions, counterbalancing uPA and plasmin actions [23, 24]. Notably, PAI-1 is not expressed in normal kidney glomeruli but is upregulated in situations of podocyte stress, like uPAR [18, 25]. PAI-1 interacts with uPAR accelerating podocyturia via the *α*_3_ *β*_1_ integrin, the main contributor of tight adhesion of podocytes to the glomerular basement membrane, and also through the *α*_*v*_ *β*_3_ integrin route [26, 27]. Therefore, uPAR-activated podocytes via the above-mentioned integrins result in the effacement of foot processes through disruption of the actin cytoskeleton [25]. This mechanism could trigger podocyturia, being potentiated by PAI-1 [25].

Finally, developmental and homeostatic remodeling of the extracellular matrix is a highly regulated process orchestrated by a family of zinc-containing, calcium-dependent, secreted neutral proteases known as the matrix metalloproteinases (MMPs). This family of enzymes can collectively degrade all structural proteins including basement membrane collagens (IV), fibronectin, laminin, proteoglycans, and elastin. The MMPs include collagenases, gelatinases, and atrilysin. Elevated expression of MMP-9 is observed in fibrotic renal cortex from X-linked Alport syndrome dogs. These findings suggest that MMPs may play an important role in matrix accumulation associated with progressive renal scarring in this model [28]. Although not specifically investigated in Alport syndrome, in vitro keratinocyte cell migration towards fibronectin was documented to be stimulated by plasminogen and plasmin by a MMP-9 dependent mechanism and cooperatively interacting with *β*1 integrins. Noteworthily, the addition of the uPA inhibitor amiloride decreased cell migration, probably by decreasing plasmin levels [17, 29].

In summary, the decrease in podocyturia displayed by our patient after amiloride administration may suggest that the above-mentioned podocyte molecular pathways may be involved in the pathogenesis of podocyturia and the development of proteinuria in Alport syndrome. Due to the lack of specific treatments, amiloride may prove a useful, nonexpensive drug to be employed in Alport syndrome in order to delay kidney disease progression, mainly by the preservation of the podocyte population.
